# The Novelty-Seeking Phenotype Modulates the Long-Lasting Effects of Intermittent Ethanol Administration during Adolescence

**DOI:** 10.1371/journal.pone.0092576

**Published:** 2014-03-21

**Authors:** Sandra Montagud-Romero, Manuel Daza-Losada, Antonio Vidal-Infer, Concepción Maldonado, María A. Aguilar, Jose Miñarro, Marta Rodríguez-Arias

**Affiliations:** Unidad de Investigación Psicobiología de las Drogodependencias, Departamento de Psicobiología, Facultad de Psicología, Universitat de València, Valencia, Spain; Roma Tre University, Italy

## Abstract

The aim of the present study was to investigate if a novelty-seeking phenotype mediates the long-lasting consequences of intermittent EtOH intoxication during adolescence. The hole board test was employed to classify adolescent mice as High- or Low-Novelty Seekers. Subsequently, animals were administered ethanol (1.25 or 2.5 g/kg) on two consecutive days at 48-h intervals over a 14-day period. Anxiety levels - measured using the elevated plus maze- spontaneous motor activity and social interaction test were studied 3 weeks later. A different set of mice underwent the same procedure, but received only the 2.5 g/kg dose of ethanol. Three weeks later, in order to induce CPP, the same animals were administered 1 or 6 mg/kg of cocaine or 1 or 2.5 mg/kg MDMA. The results revealed a decrease in aggressive behaviors and an anxiolytic profile in HNS mice and longer latency to explore the novel object by LNS mice. Ethanol exposure enhanced the reinforcing effects of cocaine and MDMA in both groups when CPP was induced with a sub-threshold dose of the drugs. The extinguished cocaine-induced CPP (1 and 6 mg/kg) was reinstated after a priming dose in HNS animals only. Our results confirm that intermittent EtOH administration during adolescence induces long-lasting effects that are manifested in adult life, and that there is an association between these effects and the novelty-seeking phenotype.

## Introduction

Many reports indicate that alcohol is one of the first drugs of choice among young people and adolescents and that heavy binge-drinking is increasingly frequent among this age group in numerous countries [1], [2], [3]. A “binge” is a pattern of alcohol consumption that results in the blood's alcohol concentration reaching 0.08-gram percent or above; i.e. 5 or more drinks in about 2 hours in the case of a typical male, and 4 or more drinks consumed in a similar period of time in the case of a typical female [3], [4], [5]. The brain is one of the major targets of the actions of ethanol (EtOH), and heavy alcohol consumption produces significant alterations of the structure, physiology and function of this organ [6]. Concerns about heavy drinking during adolescence - a period of brain maturation - have grown since recent evidence demonstrated that it can have a negative impact on brain structure and function, causing significant short- and long-term cognitive and behavioral consequences [7]. Indeed, these damaging effects of EtOH are usually irreversible [8]. Episodic alcohol intoxication or binge-type administration of alcohol to experimental animals increases cell death in the neocortex, hippocampus and cerebellum [9], [10], [11]. Furthermore, animals exposed to EtOH during adolescence exhibit behavioral deficits that linger after treatment and into adulthood [12], [13]. On the other hand, studies in rodents have shown that adolescent animals are less sensitive to the negative consequences of alcohol on motor impairment [14] or in terms of hangover effects [15], but are more sensitive than adults to some of its pleasurable effects, including social facilitation [16].

Drug-taking early in life is associated with an increased rate of drug abuse and dependence in later years [17]. However, not all users progress to dependence, and research suggest that this transition depends on certain traits. For example, high impulsivity and/or sensation-seeking are thought to make individuals particularly susceptible [18], [19]. During adolescence, humans and animals undergo numerous behavioral changes, including an increase in risk-taking, novelty-seeking and sensation-seeking behavior [20]. Such behaviors facilitate the adolescent's transition to maturity [21] and provide adaptive benefits by promoting independence and the ability to survive in the absence of parental guidance. In some adolescents, risk-taking behaviors may even be a strategy to combat dysphoria or to cope with stress [22], [23], [24]. However, these behaviors can leave adolescents more vulnerable to harm, as many perceive novel experiences involving drugs and alcohol to be rewarding and exciting [20]. Animal studies also point to the dominance of rewarding versus aversive effects of addictive drugs during adolescence [17], [25], [26], [27], [28]. Consequently, the traits of novelty- and sensation-seeking are significantly stronger in drug users than in non-users [29]. This ‘willingness to take risks’ and the ‘active search for sensations’ are possibly due to low levels of novelty- and risk-induced anxiety.

Novelty-seeking in rodents can be defined as the enhanced specific exploration of novel situations, unknown objects or stimuli. It is a complex behavior that involves the detection of changes in the environment (cognition) and is related to stress responsiveness [30]. Rodents naturally tend to approach and explore novel objects, showing an innate preference over familiar ones [31]. The behavior of animals in novel environments is conditioned by the interaction of several factors, including activity, motivation to explore, and fear/anxiety [32], [33]. Different procedures have been used to screen rats and mice according to either high or low expression of a given measure. The hole-board test evaluates propensity to explore a new environment in a free-choice procedure. This test, developed in 1962–1964 by Boissier and Simon, provides a simple means with which to assess the response of an animal to an unfamiliar setting [34]. “Head-dipping”, the exploratory behavior measured in this test, is considered to represent exploratory tendencies that are distinct from general locomotor activity and has been proposed as a useful measure of the relationship between novelty-seeking and drug abuse [35].

High-sensation seekers are more likely to experiment with illicit drugs [18], [36], are more responsive to drugs of abuse - as measured by various scales of the Addiction Research Center Inventory (ARCI), Profile of Mood States (POMS) and Visual Analogue Scale (VAS), which quantify positive subjective effects [19], [37] - and display greater incentive motivation to self-administer amphetamine in a laboratory setting [37]. In animal models, rats that are more responsive to novelty have been shown to acquire cocaine self-administration with a shorter latency [38] and to develop CPP with a sub-threshold dose of this drug [13].

A relationship has been highlighted between ethanol and novelty-seeking. Ethanol's positive effects are enhanced in individuals with sensation-seeking personalities [39]. Moreover, novelty-seeking is associated with increased alcohol intake, alcoholism and relapse, suggesting that it predisposes individuals to alcohol-related problems [40]. On the other hand, in experimental animals, chronic EtOH exposure during adolescence increases the tendency of animals to engage in more exploratory or novelty-seeking behaviors [41]. Thus, the aim of the present study was to investigate if the long-lasting consequences of intermittent EtOH intoxication during adolescence are influenced by the novelty-seeking phenotype. The hole board test was employed to classify adolescent mice as High- or Low-Novelty Seekers (HNS or LNS, respectively). The animals were subsequently exposed to EtOH (1.25 or 2.5 g/kg) on two consecutive days at 48-h intervals over a 14-day period. Postnatal day (PND) 28 to 42 is considered a conservative age range during which mice are expected to exhibit neurobehavioral characteristics typical of adolescence [26]. Anxiety levels and spontaneous motor activity, measured using the elevated plus maze (EPM) and social interaction test, were studied 3 weeks after administration of EtOH. A different set of mice underwent the same procedure, but were administered only the 2.5 g/kg dose of EtOH. Three weeks later these animals were given a subthreshold dose of cocaine (1 mg/kg) or MDMA (1 mg/kg) in order to induce CPP and thus evaluate their sensitivity to the conditioned reinforcing effects of these drugs. CPP has become an alternative to self-administration for assessing the rewarding effects of addictive drugs [42], [43]. In this paradigm, the amount of time spent by drug-free animals in an environment previously paired with the drug's effects represents the rewarding or incentive properties of that drug. This, this test is a useful tool for evaluating the sensitivity of individuals to the incentive properties of addictive drugs. A large number of studies have reported that the response to a novel environment is positively correlated with the ability to acquire cocaine self-administration behavior [44], [45], [46], [47]. In line with this research, high levels of novelty-seeking have been shown to predict greater sensitivity to the conditioned rewarding effects of cocaine [13], although no studies have yet been performed with MDMA. Taking into account this previous evidence, our hypothesis was that mice with high-novelty seeking scores would be more vulnerable to the long-lasting effects of EtOH.

## Materials and Methods

### Subjects

We employed a total of 251 male mice of the OF1 strain (CHARLES RIVER, Barcelona, Spain) in this study. The mice were 21 days old on arrival at the laboratory and were all housed under standard conditions in groups of four (cage size 28×28×14.5 cm), at a constant temperature (21+2°C), with a reversed light schedule (white lights on 19:30–07:30 h) and food and water available ad libitum (except during the behavioral test). All procedures involving the mice and their care complied with national, regional and local laws and regulations, and with European Community Council Directives (86/609/EEC, 24 November 1986). The protocol was approved by the Committee on the Ethics of Animal Experiments of the University of Valencia (Permit Number: A1364467956525).

### Drugs

For chronic EtOH exposure, animals were injected i.p. with 1.25 or 2.5 g/kg of EtOH in a volume of 0.02 ml/g (the higher dose) or 0.01 ml/g (the lower dose). The control group was injected with the physiological saline (NaCl 0.9%) used to dissolve the drugs. For place conditioning, animals were injected i.p. with 1 or 6 mg/kg of cocaine hydrochloride (Laboratorios Alcaliber S. A. Madrid, Spain) and 1 or 2.5 mg/kg of MDMA (±3,4-methylene-dioxy-metamphetamine hydrochloride, Laboratorios Sigma-Aldrich, Spain) in a volume of 0.01 ml/g. The dose selected to induce CPP was based on previous studies [48], [49], [50], [51], [52].

### Experimental design and drug pre-exposure

After an acclimatization period of 10 days (PND 31), adolescent animals performed the hole board test and were defined as HNS or LNS according to their head dip scores (below or above the median).

Animals received 16 doses of EtOH (1.25 or 2.5 g/kg) or saline over a 2-week period according to the following schedule: twice daily administrations (with a 4-hour interval) on two consecutive days separated by an interval of 2 days during which no injections were administered. Animals were injected on PND 33, 34, 37, 38, 41, 42, 45, and 46. In this way, each mouse received 16 drug administrations that simulated a binge pattern such as that engaged in by human adolescents and young adults [53], [54]. Behavioral tests were performed on postnatal day 67), three weeks after exposure had finalized. A separate set of mice was employed for the CPP studies and underwent a similar exposure schedule in which only the dose of 2.5 g/kg of EtOH was administered. A more detailed description of the experimental procedure is provided in Table 1.

**Table 1:** Experimental design.

PND	31	33–34	35–36	37–38	39–40	41–42	43–44	45–46	47–66
	Hole-Board	EtOH/Sal		EtOH/Sal		EtOH/Sal		EtOH/Sal	No treatment
		9:00–13:00		9:00–13:00		9:00–13:00		9:00–13:00	

PND	67	68	69	70	71	72	73	74	75	76	77	78
	EPM		2nd Hole Board	Novel object	Social Inte-raction							
	CPP: Pre-C test			Cocaine -induced CPP: conditioning				CPP: Post-C test				
	CPP: Pre-C test			MDMA -induced CPP: conditioning								CPP: Post-C test

### Hole board

The hole board consisted of a box (28×28×20.5 cm) made of clear Plexiglas and with 16 equidistant holes with a diameter of 3 cm in the floor and walls (CIBERTEC, SA, Spain). Photocells below the surface of the holes detected the number of times a mouse engaged in head dipping. Each hole was numbered so that the number of times a mouse explored a specific hole could be counted. At the beginning of each test, the mouse was placed in one corner of the hole-board and allowed to explore it freely for 10 min. The behaviors scored were latency times for performing the first head dip and total number of dips in 10 min. The mice performed this test on PND 31 and PND 69.

### Social interaction test

This test consisted of confronting an experimental animal with a standard opponent in a neutral cage (61×30.5×36 cm) for 10 min following a 1-min adaptation period prior to the encounter. On the day before testing standard opponents were rendered temporarily anosmic by intranasal lavage with a 4% zinc sulfate solution [55]. This kind of mouse induces an attack reaction in its opponent but does not outwardly provoke or defend itself, since it cannot perceive a pheromone present in the urine of the experimental animal that functions as a cue for eliciting aggressive behavior in mice with a normal sense of smell [56], [57]. The animals' behavior was videotaped under white illumination and the videotapes were subsequently analyzed using a custom-developed program [58] that facilitates the estimation of times allocated to different broad functional categories of behavior – body care, digging, non-social exploration, social investigation, threat and attack - each of which is characterized by a series of different postures and elements. A more detailed description can be found in Rodríguez-Arias et al [59].

### Elevated plus maze

The EPM consisted of two open arms (30×5×0.25 cm) and two enclosed arms (30×5×15 cm), and the junction of the four arms formed a central platform (5×5 cm). The floor of the maze was made of black Plexiglas and the walls of the enclosed arms were made of clear Plexiglas. The open arms had a small edge (0.25 cm) to provide additional grip for the animals. The entire apparatus was elevated 45 cm above floor level. In order to facilitate adaptation, mice were transported to the dimly illuminated laboratory 1 h prior to testing. At the beginning of each trial, subjects were placed on the central platform so that they were facing an open arm and were allowed to explore for 5 min. The maze was thoroughly cleaned with a damp cloth after each trial. The behavior displayed by the mice was video recorded and later analyzed by a ‘blind’ observer using a computerized method. The measurements recorded during the test period were number of entries and time and percentage of time spent in each section of the apparatus (open arms, closed arms, central platform). An arm was considered to have been visited when the animal placed all four paws on it. Number of open arm entries, time spent in open arms, and percentage of open arm entries are generally used to characterize the anxiolytic effects of drugs [60], [61].

### Novel object recognition task

Procedures were similar to those described by Frick and Gresack [62]. Single trial novel object recognition was performed in an open arena (24×24 cm) using 2 object types (two small stones and a small color toy) that were fixed with Velcro tape to the floor at opposite corners of the open field, 5 cm from the walls. Each mouse completed a daily trial on four successive days. In trial 1 (habituation phase), the mouse was placed in the center of the empty open field box and allowed to explore it freely. During trials 2 and 3 (sample phase), two of the stones were placed in opposite corners of the box and the mouse was allowed to explore them. In trial 4 (test phase), one of the objects was replaced with a new object in order to assess novel object exploration. Each trial lasted 10 min. Only the first 5 min of the test were analyzed. The box and objects were carefully cleaned with 70% alcohol at the end of each trial. Object exploration was defined as intentional contact between the mouse's nose or legs and the novel object. Behaviors scored were: (a) latency times for making contact with the novel object; (b) percentage of time spent exploring the novel object; and (c) number of explorations of the novel object.

### Conditioning place preference

For place conditioning, we employed eight identical Plexiglas boxes with two equal size compartments (30.7 cm length ×31.5 cm width ×34.5 cm height) separated by a gray central area (13.8 cm, length ×31.5 cm, width ×34.5 cm height). The compartments have different colored walls (black vs white) and distinct floor textures (fine grid in the black compartment and wide grid in the white one). Four infrared light beams in each compartment of the box and six in the central area allowed the recording of the position of the animal and its crossings from one compartment to the other. The equipment was controlled by two IBM PC computers using MONPRE 2Z software (CIBERTEC, SA, Spain).

Place conditioning consisted of three phases and took place during the dark cycle, during which animals were allowed access to both compartments of the apparatus for 15 minutes (900 seconds) per day over 3 days (PND 67–69). On day 3, the time spent by an animal in each compartment during a period of 900 seconds was recorded. Half the animals in each group received the drug or vehicle in one compartment, and the other half received it in the other compartment. An analysis of variance revealed that there were no significant differences between the time spent in the drug-paired and vehicle-paired compartments during the Pre-C phase. In the CPP induced by cocaine (1 and 6 mg/kg), the animals underwent two pairings per day: those conditioned with cocaine received an injection of physiological saline before being confined to the vehicle-paired compartment for 30 min, and, after an interval of 4 h, received cocaine immediately before confinement to the drug-paired compartment for 30 min (PND 70–73). In the CPP induced by MDMA (1 and 2.5 mg/kg), mice underwent only one pairing per day: animals conditioned with MDMA received an injection of MDMA immediately before confinement to the drug-paired compartment for 30 min on days 4, 6, 8 and 10 (PND 70, 72, 74 and 76) and received physiological saline before being confined to the vehicle-paired compartment for 30 min on days 5, 7, 9 and 11 (PND 71, 73, 75 and 77). The central area was never used during conditioning and was blocked by guillotine doors.

During the third phase, or post-conditioning (Post-C), which took place on PND 74 (cocaine-induced CPP) or PND 78 (MDMA-induced CPP), the guillotine doors separating the two compartments were removed and the time spent by the untreated mouse in each compartment was recorded during a 900-s observation period (Post-C tests were performed between 10:00 and 14:00 h). The difference in seconds between the time spent in the drug-paired compartment in the Post-C test and Pre-C test is a measure of the degree of conditioning induced by the drug. If this difference is positive, then the drug has induced a preference for the drug-paired compartment, whereas the opposite indicates the induction of an aversion.

The groups that developed CPP underwent an extinction session every 72 h during which animals were placed in the apparatus (without the guillotine doors separating the compartments) for 15 min until the time spent in the drug-paired compartment by each group was similar to that of Pre-C. In this way, all the animals in each group underwent the same number of extinction sessions, independently of their individual scores, as the criterion for extinction was a lack of significant differences with respect to Pre-C values. The extinction of CPP was always confirmed in a subsequent session performed 24 h after the last extinction session. The effects of a priming dose (cocaine or MDMA) were evaluated 24 h after confirmation of extinction. Reinstatement tests (performed between 10:00 and 14:00 h) were the same as for Post-C (free ambulation for 15 min), except that animals were tested 15 min after administration of the respective dose of drug employed during the conditioning phase (cocaine or MDMA).

Similar procedures were employed for the groups conditioned with 6 mg/kg of cocaine or 2.5 mg/kg of MDMA. These mice performed the hole board test on PND 28 and were subsequently classified as H- or LNS, The corresponding CPP was initiated on PND 35.

### Statistical analyses

Data for the plus maze, social interaction test and novel object recognition task were analyzed by two-way ANOVA with two between factors: “exposure”, with three levels (saline, EtOH 1.25 mg/kg and EtOH 2.5 mg/kg), and “novelty seeking”, with two levels (High NS and Low NS).

The data for the number of Dips in the hole board test were analyzed with a three-way ANOVA with repeated measures and with two between factors - “exposure”, with two levels (saline and EtOH 2.5 mg/kg), and “Novelty-seeking”, with two levels (High and Low)- and a within factor - “Days”, with two levels (First HB and Second HB).

The data for the time spent in the drug-paired compartment in Pre-C and Post-C were analyzed with a three-way ANOVA with repeated measures with two between factors - “exposure”, with two levels (saline and EtOH 2.5 mg/kg), and “Novelty-seeking”, with two levels (High and Low) - and a within factor -“Days”, with two levels (Pre-C and Post-C). Differences between the time spent by mice in the drug- and saline-paired compartments in extinction and reinstatement tests after receiving priming doses were analyzed by means of a Student's “t” test. The effect of the novelty-seeking phenotype on the CPP induced by the highest doses of cocaine or MDMA was analyzed with a within factor: “Days”, with four levels (Pre-C, Post-C, extinction and reinstatement). Post hoc comparisons were performed with Bonferroni tests.

## Results

### Hole board

**Figure 1:**
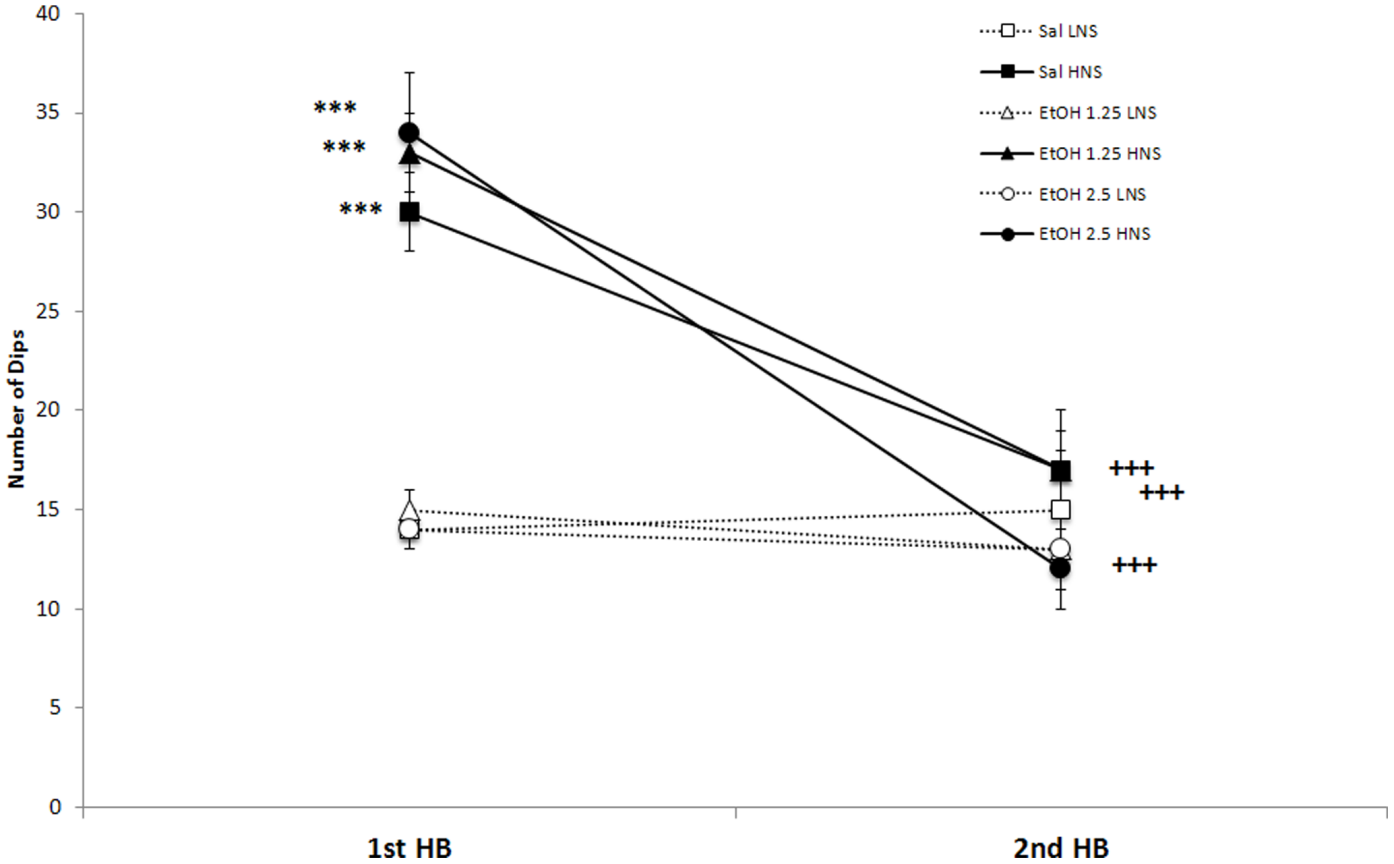
Effect of adolescent chronic intermittent ethanol exposure on novelty seeking behavior of mice according to number of head dips in the hole-board test. The significantly difference in the number of dips between high novelty seekers (HNS) and low novelty seekers (LNS) observed in adolescent mice (PND 31) (p<0.001) are no longer presented in adult mice (PND 69). HNS adult mice, treated with saline or EtOH, significantly reduced the number of dips in comparison to the number of dips presented before treatment (p<0.001). ***p<0.001, significant differences with respect LNS; +++p<0.001, significant differences with respect HNS in the 1^st^ HB test.

The number of dips performed in the hole board test (see Figure 1) revealed an effect of the factor Novelty-Seeking [F(1,88) = 66.088; p<0.001], with HNS performing more Dips that their LNS counterparts (p<0.001). The factor Days [F(1,88) = 48.4889; p<0.001] and the interaction Days × Novelty-seeking [F(1,88) = 40.001; p<0.001] also had significant effects, with HNS mice performing more Dips in the first HB test than in the second (p<0.001). In addition, HNS performed significantly more Dips in the first HB than in the second (p<0.001). No differences were observed among LNS between the first and second HB test.

### Social interaction test

The data for the behaviors evaluated in the social interaction test are presented in Table 2. The factor Novelty-Seeking showed a significant effect of Body Care [F(1,84) = 5,356; p<0.02] and Non-Social Exploration [F(1,84) = 5,660; p<0.02]. HNS devoted more time to Body care than LNS (p<0.02). However, LNS spent more time in Non-Social exploration than HNS (p<0.02). These effects were significant with respect to the control group (p<0.05 and p<0.01, respectively), but no significant differences were observed among the EtOH-exposed groups. The factor Exposure showed a significant effect on Non-Social exploration [F(2,84) = 3,183; p<0.05], Threat [F(2,84) = 3,920; p<0.02] and Attack [F(2,84) = 4,077; p<0.02]. Mice exposed to the highest EtOH dose spent more time engaged in Non-Social exploration than controls, but this increase was observed only in HNS (p<0.01). Equally, the highest EtOH dose significantly decreased Threat and Attack behavior with respect to controls in HNS mice only (P<0.02 and p<0.03, respectively).

**Table 2:** Effects of intermittent ethanol administration during adolescence on the time engaged in different types of spontaneous behavior during the social interaction test.

	Sal		EtOH 1.25		EtOH 2.5	
	LNS	HNS	LNS	HNS	LNS	HNS
**Body care**	13±2	30±1	15±5	27±7	12±4	14±4
**Digging**	8±2	8±2	6±1	7±1	7±1	10±2
**Non-social exploration**	481±12	403±3	486±13	457±11	488±11	489±16**
**Explore from a distance**	13±2	18±1	11±5	12±6	13±1	12±2
**Social investigation**	72±10	73±1	73±7	88±10	78±8	74±14
**Threat**	8±4	20±1	6±5	5±4	0±0	0±0*
**Attack**	4±2	9±1	3±2	3±2	0±0	0±0*

Means of accumulated times (in seconds) with ±S.E.M. devoted to each category of spontaneous behavior by H- and LNS mice exposed during adolescence to ethanol (Sal, 1.25 or 2.5 g/kg). The social interaction test was performed on PND 71.

*p<0.05,

**p<0.01 significant difference with respect to HNS saline-exposed group.

### Elevated plus maze

The ANOVA revealed an effect of the factor Exposure and the interaction Exposure × Novelty Seeking on time [F(2,76) = 4.487; p<0.01]; [F(2,76) = 3.184; p<0.05] and percentage of time spent in the open arms of the maze [F(2,76) = 4.492; p<0.01], [F(2,76) = 3.171; p<0.05]. EtOH significantly increased the time and percentage of time spent in the open arms with respect to control groups in HNS mice only (p<0.01 in all cases). The factor Exposure also had an effect on the number of open entries [F(2,76) = 3.335; p<0.04], with control mice performing more entries into the open arms that those treated with the higher dose of EtOH (p<0.01) (see Table 3).

**Table 3:** Long-term effects of adolescent chronic intermittent ethanol exposure on performance in the elevated plus maze.

	Saline		EtOH 1.25		EtOH 2.5	
	LNS	HNS	LNS	HNS	LNS	HNS
**Time OA**	45±8	31±10	53±12	76±16**	56±11	75±9**
**% Time OA**	15±3	10±3	18±4	25±5**	19±4	25±4**
**Time Center**	174±8	178±14	176±13	151±18	171±12	135±15
**Time in CA**	81±8	92±12	71±7	74±8	73±8	89±10
**Open Entries**	4±3	3±1	5±1	5±1	5±1**	7±1**
**% Open Entries**	15±3	13±3	17±3	20±4	17±3	23±3
**Closed Entries**	10±1	11±2	9±1	8±1	9±3	10±3
**Total entries**	28±2	29±3	28±2	27±2	8±3	34±5

Data are presented as mean values ± S.E.M. Mice performed the elevated plus maze test on PND 67.

**p<0.01 significant difference with respect to the corresponding saline-exposed group.

### Novel object recognition task

The percentage of time spent exploring the novel object [F(2,85) = 9.060; p<0.001] and the number of explorations [F(2,85) = 3.055; p<0.05] were affected by the factor Exposure. Both EtOH doses induced a decrease in the percentage of time spent exploring the novel object (p<0.01 for EtOH 1.25 and p<0.001 for EtOH 2.5). On the other hand, the higher EtOH dose reduced the number of explorations in comparison with controls (p<0.05). The latency for making contact with the novel object showed an effect of the interaction Exposure × Novelty-Seeking [F(2,85) = 2.916; p<0.05]. The higher EtOH dose significantly increased the time needed to make the first contact with respect to controls in LNS mice only (p<0.01). In addition, LNS animals exposed to this dose of EtOH presented longer latencies to explore the novel object than HNS animals exposed to the same dose (p<0.05) (see Table 4).

**Table 4:** Long-term effects of adolescent chronic intermittent ethanol exposure on performance in the novel object recognition task.

	Sal		EtOH 1.25		EtOH 2.5	
	LNS	HNS	LNS	HNS	LNS	HNS
**% Time Exploring NO**	10±3	4±1	3±0.7**	2.4±0.6	1,5±0.3**	1.8±0.5
**Latency to explore NO**	4±1	8±1.4	8±2	7±2	11±2** +	6±1
**Number of explorations NO**	29±2	27±2	27±2	26±2	22±1**	18±2**

The data represent the mean (± standard error) of the percentage of time spent exploring the novel object.

*p<0.05;

**p<0.01;

***p<0.001 significant difference with respect to the corresponding saline-exposed group;

+ p<0.05 significant difference with respect to HNS animals in the same conditions.

### Conditioning place preference induced by cocaine

**Figure 2:**
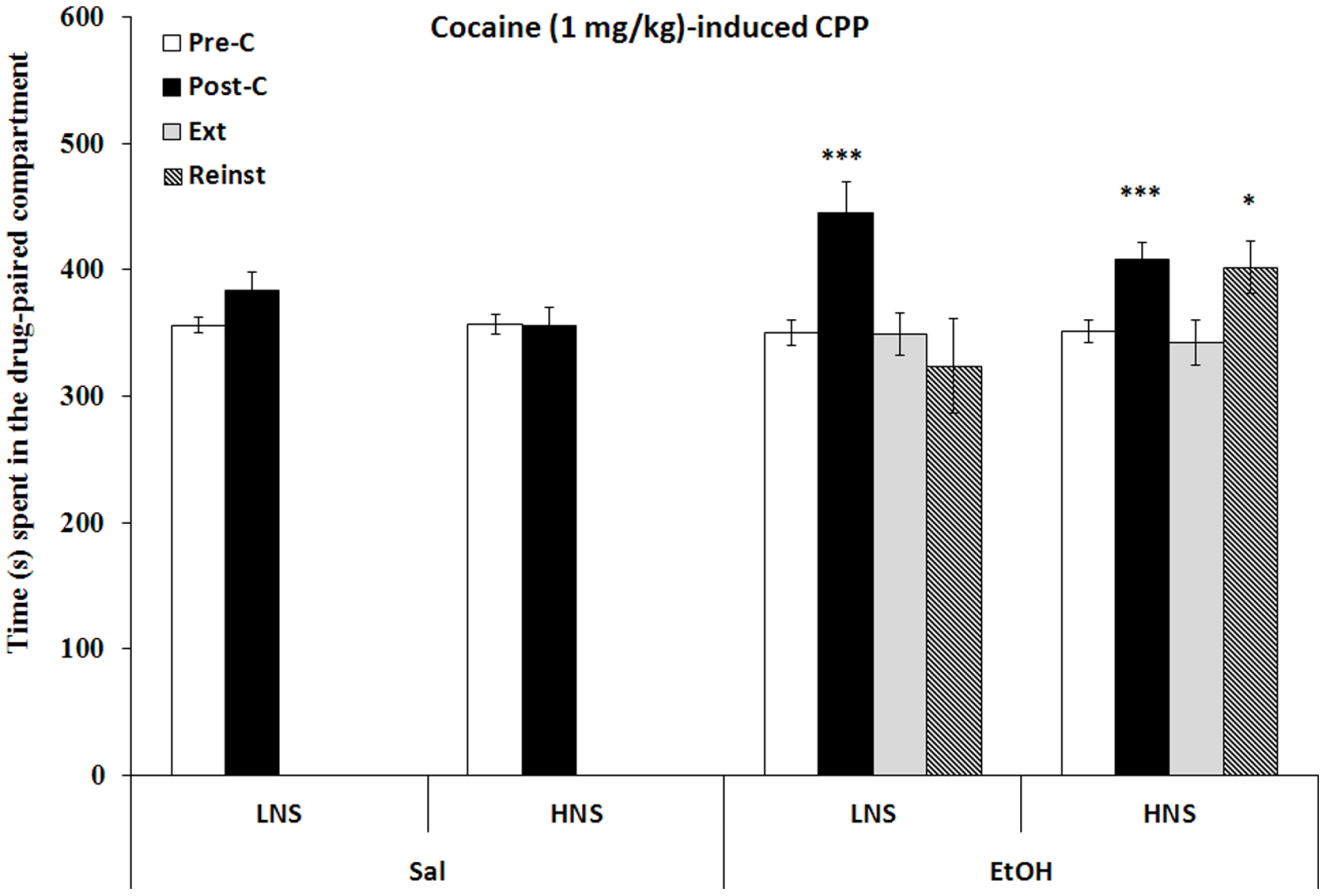
Long-term effects of adolescent chronic intermittent ethanol exposure (2.5 g/kg) on cocaine-induced CPP (1 mg/kg). Bars represent the mean (± SEM) time spent in the drug-paired compartment before conditioning sessions (white bars), after conditioning sessions (black bars), during the last extinction session (light gray bars) and during the reinstatement test (black striped bars). The reinstatement test was evaluated 15 mins after a priming dose of 0.5 mg/kg of cocaine. *p<0.05; ***p<0.001 significant difference in the time spent in Post-C vs Pre-C sessions.

The results regarding the effects of EtOH on 1 mg/kg of cocaine-induced CPP are presented in Figure 2. The ANOVA revealed a significant effect of the factors Days [F (1,47) = 18.158;p<0.001] and Exposure [F(1,47) = 5.384;p<0.01] and the interaction Days × Exposure [F(1,47) = 8.978; p<0.01]. The EtOH-exposed groups spent more time in the drug-paired compartment in the Post-C test than in the Pre-C test (p<0.001). Moreover, mice exposed to EtOH spent more time in the drug-paired compartment in Post-C than saline-exposed controls (p<0.01). HNS EtOH-exposed mice required 8 sessions for the preference to be extinguished, while LNS EtOH-exposed mice needed only 4 sessions. A priming dose of 0.5 mg/kg of cocaine reinstated the preference in the HNS EtOH-group only (p<0.02), but no reinstatement was observed in the LNS EtOH-exposed group.

**Figure 3:**
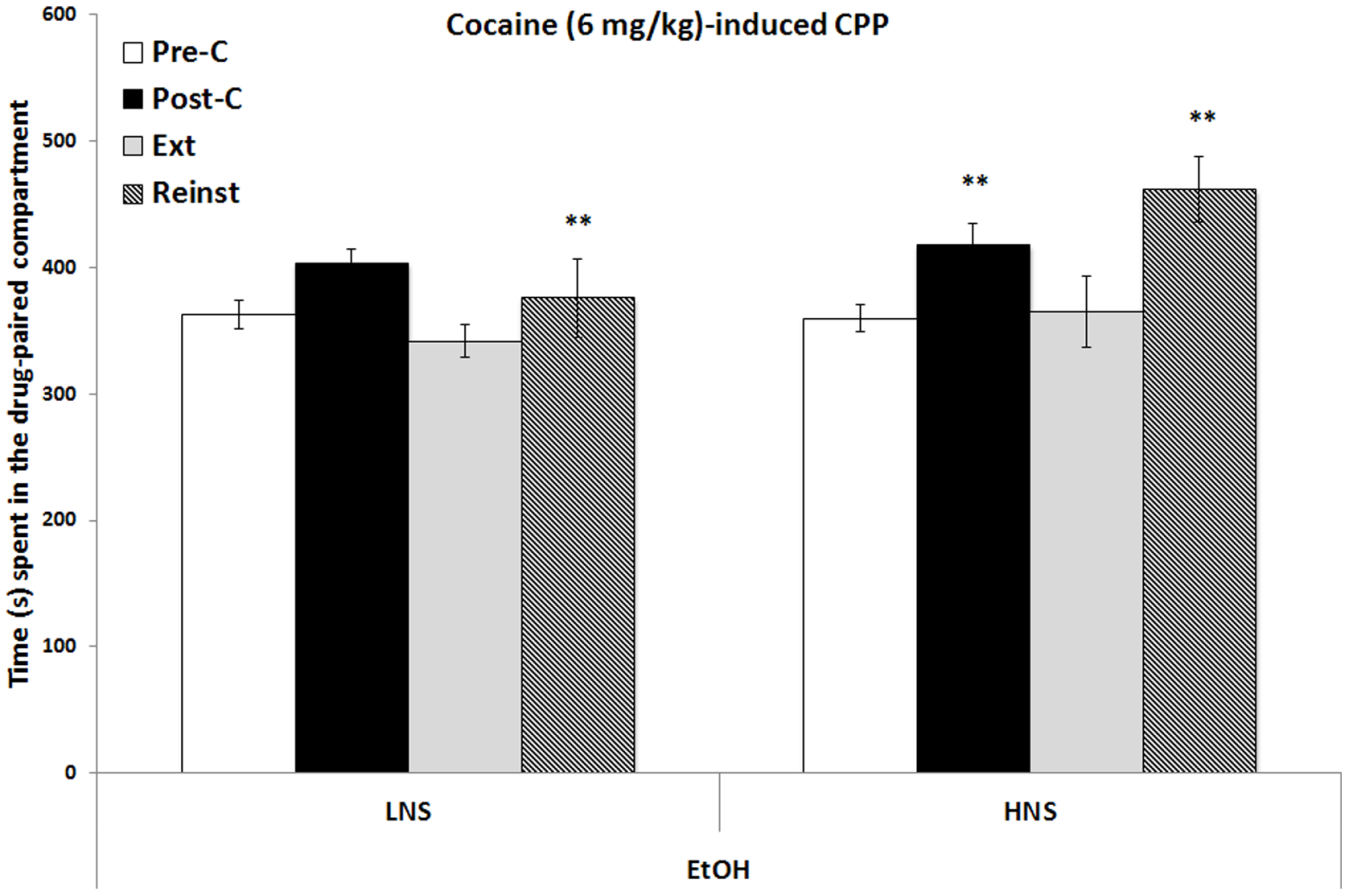
Long-term effects of adolescent chronic intermittent ethanol exposure (2.5 g/kg) on cocaine (6 mg/kg) - induced CPP. Bars represent the mean (±SEM) time spent in the drug-paired compartment before conditioning sessions (white bars), after conditioning sessions (black bars), during the last extinction session (light gray bars) and during the reinstatement test (bars with black stripes). The reinstatement test was evaluated 15 mins after a priming dose of 3 mg/kg of cocaine or 1.25 mg/kg of MDMA. ** p<0.001 significant difference in the time spent in Pre-C vs Post-C sessions or reinstatement test vs extinction.

The results regarding the CPP induced by 6 mg/kg of cocaine in H- and LNS mice are presented in Figure 3. The ANOVA revealed a significant effect of the factor Days [F (3,60) = 6.544;p<0.001] and the interaction Days × Novelty Seeking [F(3,60) = 2.207; p<0.05]. H- and LNS mice developed cocaine-induced CPP, as they spent more time in the cocaine-paired compartment in the Post-C test than in the Pre-C test (p<0.01). HNS mice required 19 extinction sessions for the preference to be extinguished and LNS mice required 12 extinction sessions. Once extinction had been achieved, a priming dose of 3 mg/kg of cocaine reinstated the preference in HNS mice only (p<0.01).

### Conditioning place preference induced by MDMA

**Figure 4:**
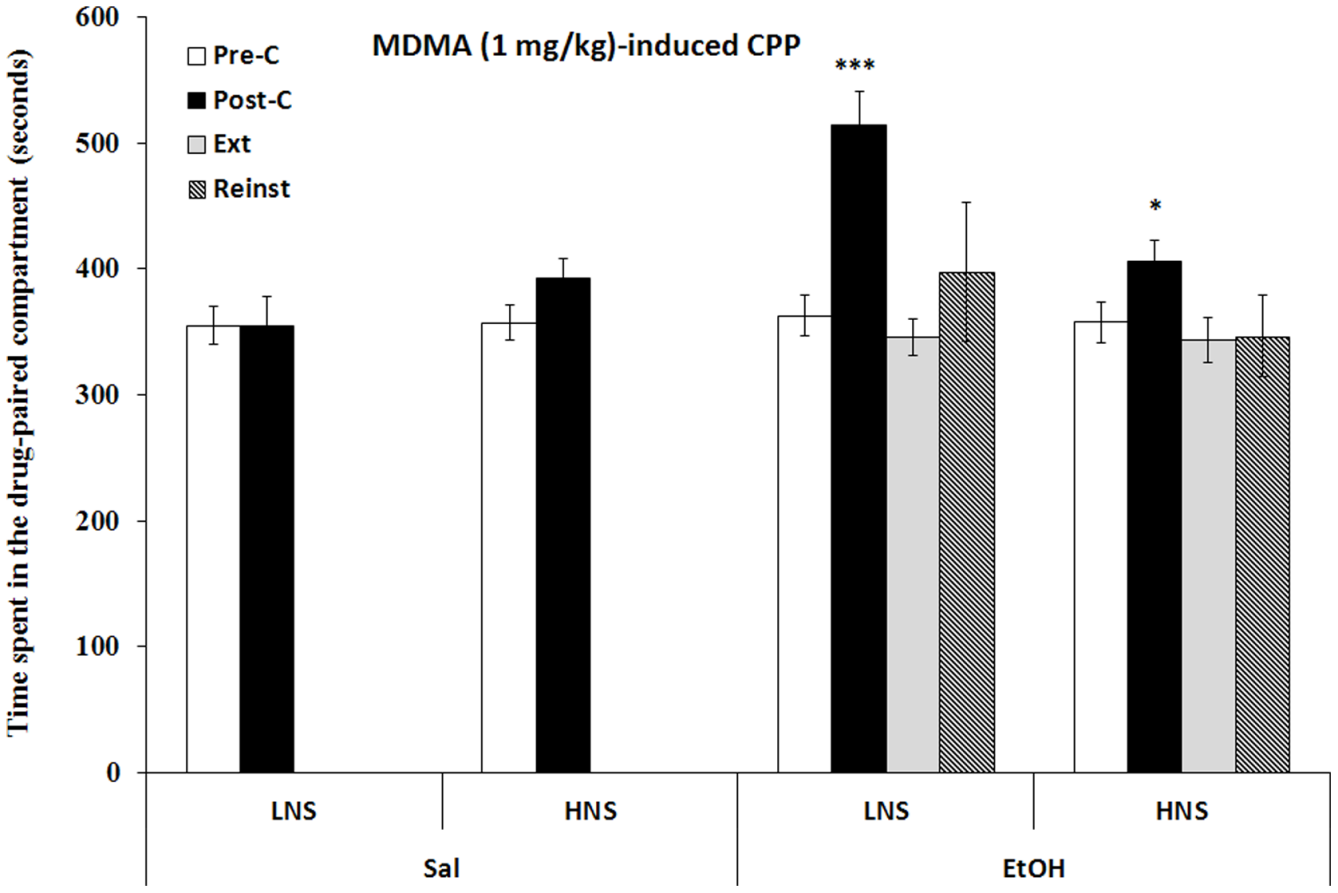
Long-term effects of adolescent chronic intermittent ethanol exposure (2.5 g/kg) on MDMA-induced CPP (1 mg/kg). Bars represent the mean (7 SEM) time spent in the drug-paired compartment before conditioning sessions (white bars), after conditioning sessions (black bars), during the last extinction session (light gray bars) and during the reinstatement test (bars with black stripes). The reinstatement test was evaluated 15 mins after a priming dose of 0.5 mg/kg of MDMA. *P<0.05, ***P<0.001 significant difference in the time spent in Post-C vs Pre-C sessions.

The results regarding the effects of EtOH on 1 mg/kg of MDMA-induced CPP are presented in Figure 4. The ANOVA revealed a significant effect of the factor Days [F (1,42) = 33.700; p<0.001] and the interaction Days × Exposure [F (1,42) = 9.339; p<0.004], as all the EtOH-exposed groups spent more time in the drug-paired compartment in the Post-C test than in the Pre-C test (p<0.001). A significant effect was also observed with respect to the interaction Days × Novelty Seeking × Exposure [F (1,42) = 4.287; p<0.04]. LNS exposed to EtOH spent significantly more time than their HNS counterparts in the drug-paired compartment during the Post-C test (p<0.001). HNS EtOH-exposed mice required 6 sessions for the preference to be extinguished, while LNS EtOH-exposed mice needed only 7 sessions. A priming dose of 0.5 mg/kg of MDNA did not reinstate the preference in either group.

**Figure 5:**
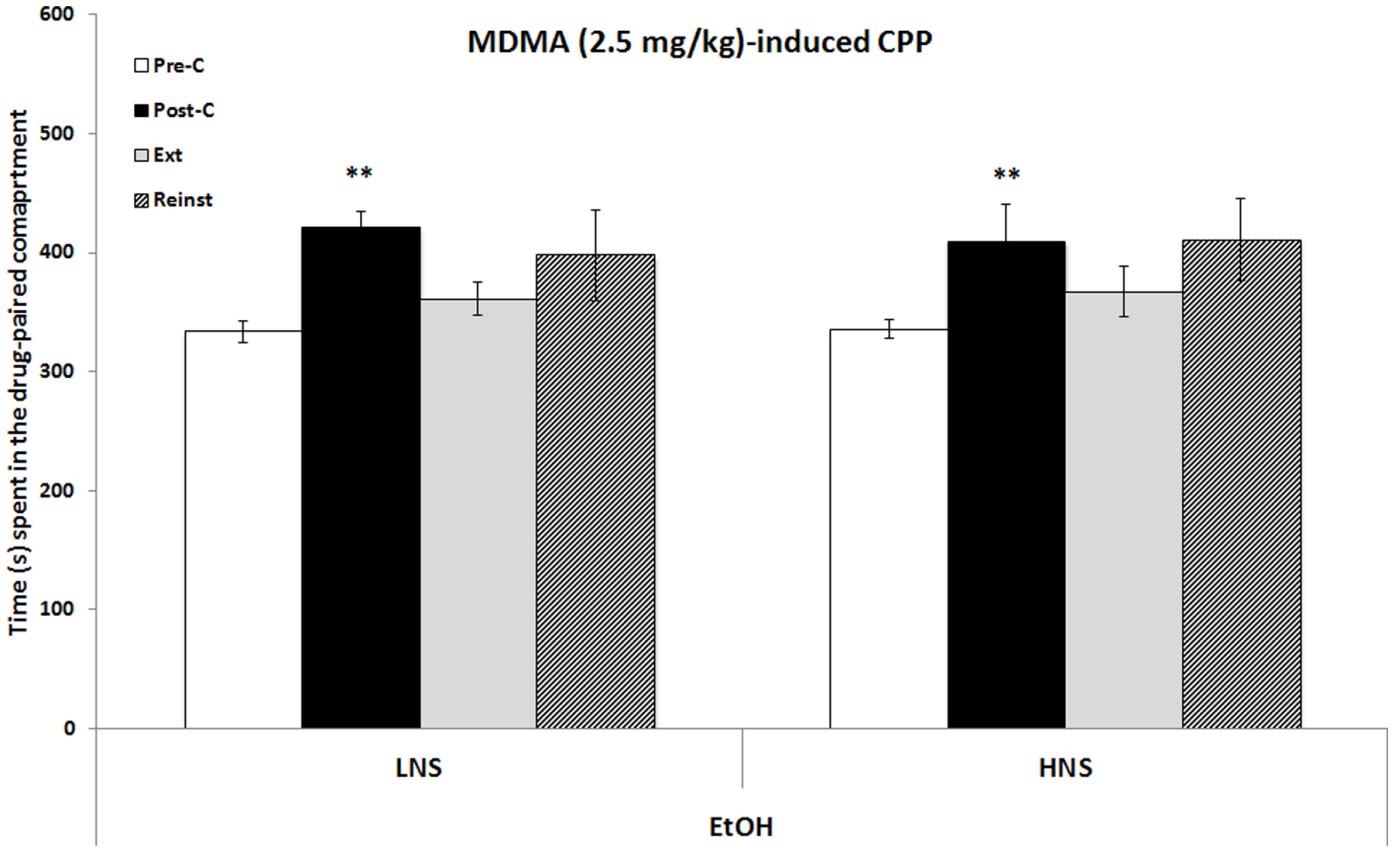
Long-term effects of adolescent chronic intermittent ethanol exposure (2.5 g/kg) on MDMA (2.5 m/kg) - induced CPP. Bars represent the mean (±SEM) time spent in the drug-paired compartment before conditioning sessions (white bars), after conditioning sessions (black bars), during the last extinction session (light gray bars) and during the reinstatement test (bars with black stripes). The reinstatement test was evaluated 15 mins after a priming dose of 3 mg/kg of cocaine or 1.25 mg/kg of MDMA. ** p<0.001 significant difference in the time spent in Pre-C vs Post-C sessions or reinstatement test vs extinction.

The results regarding the effects of 2.5 mg/kg MDMA-induced CPP on H- and LNS mice are presented in Figure 5. The ANOVA revealed a significant effect of the factor Days [F (3,87) = 6.774;p<0.001]. H- and LNS mice developed MDMA-induced CPP, as they spent more time in the cocaine-paired compartment in the Post-C test than in the Pre-C test (p<0.01). HNS mice required 11 extinction sessions for the preference to be extinguished, while LNS animals required 25. Once extinction had been achieved, a priming dose of 1.25 mg/kg of MDMA did not reinstate the preference in either group.

## Discussion

Our results confirm that EtOH binge drinking during adolescence induces long-lasting effects that continue to manifest themselves in adult life. We also demonstrate that these effects were modulated by the level of novelty-seeking of our animals. In male mice, regardless of novelty phenotype, exposure during adolescence to a binge pattern of EtOH administration produced an increase in the reinforcing effects of cocaine and MDMA, as they developed CPP with a sub-threshold dose of these drugs. However, exposure to EtOH during adolescence reduced aggressive behaviors and exerted an anxiolytic effect in HNS mice only. Moreover, cocaine-induced CPP was reinstated in these mice with the doses of 1 and 6 mg/kg when they had received a priming dose.

Few studies have evaluated the effect of pre-exposure to EtOH on the rewarding effects of cocaine, and those employing cocaine-induced CPP report discrepant results. In two studies performed in adult rats, exposure to EtOH did not increase the reinforcing effects of cocaine in the CPP [63], [64]. However, in a more recent study, adult and adolescent rats undergoing daily EtOH administration for 10 days showed an increased response to a cocaine-induced CPP 26 days after exposure had ended [65]. Our results are in line with those of that study, as mice pre-exposed to our model of binge drinking during adolescence developed cocaine-induced CPP in adulthood with a non-effective dose of cocaine (1 mg/kg). Preference developed in both H- and LNS mice, although the latter required only 4 extinction sessions for it to be extinguished, while HNS mice needed 8 sessions. Once extinction had been achieved, a priming dose of 0.5 mg/kg of cocaine induced reinstatement of preference in HNS mice only. Thus, although EtOH increased the reinforcing effects of cocaine in both H- and LNS mice, more sessions were required for the preference to be extinguished and the preference was reinstated after a priming dose of cocaine in the former. Extinction provides a measurement of the motivational properties of drugs, which are reflected by the persistence of drug-seeking behavior in the absence of the drug. Reinstatement of the extinguished preference through a priming injection of a drug is a reliable model for studying the mechanisms of drug-craving and relapse [43]. In our experiments, HNS mice showed greater motivation for the drug and were more vulnerable to relapse. When the CPP was induced by doses capable of inducing CPP but not reinstatement after administration of a 50% priming dose [48], [52], CPP was induced in H- and LNS animals. Moreover, in cocaine-conditioned HNS mice, more sessions were required for preference to be extinguished, and preference was reinstated after a priming dose of cocaine. In LNS subjects, on the other hand, preference was extinguished in fewer sessions and no reinstatement was confirmed. These results corroborate those obtained with the lower dose of cocaine, which confirm the relevance of the novelty-seeking phenotype in the effects induced by intermittent EtOH exposure. There are no previous reports concerning this subject, but in a study by Schramm-Sapyta et al. [66], rats consumed ethanol for 10 days followed by 2 days of abstinence, and were then given a choice between ethanol and water as a measure of relapse-like behavior. The novelty-seeking phenotype of the rats did not significantly predict deprivation-stimulated consumption, but the preference for ethanol significantly correlated with early consumption.

We have previously reported that this pattern of EtOH administration during adolescence increases the reinforcing effect of MDMA [67], which is confirmed by the results of the present study. Although H- and LNS EtOH-exposed mice developed CPP, the induced preference was significantly stronger in LNS than in HNS animals. These results contrast with those obtained with cocaine, in which a stronger response was observed in the latter animals. No differences were observed in the time needed to extinguish the preference or to reinstate preference after a priming dose. In the case of the CPP induced by 2.5 mg/kg of MDMA, LNS mice needed a higher number of sessions than their HNS counterparts for the preference to be extinguished, which contrasted with the result obtained in the CPP induced by cocaine. On the other hand, in the case of cocaine, HNS animals exhibited a greater motivation for the drug than LNS mice, which, in turn, showed a greater preference and motivation for MDMA. When CPP was induced by an effective dose of MDMA, both HNS and LNS mice developed preference, while reinstatement was not achieved with a priming dose of MDMA in either group. In line with this, a recent report by Bird and Schenk [68] showed that the novelty-seeking phenotype does not significantly correlate with either acquisition or drug-seeking parameters of MDMA self-administration in rats. Our results show that the novelty-seeking phenotype modulates the effects of EtOH exposure during adolescence on MDMA in a different way to that observed with cocaine. These differences might be due to the unique pharmacology of MDMA, which, unlike other drugs of abuse, preferentially enhances synaptic 5-HT when administered acutely [69]. Although the novelty response has been attributed to DAergic mechanisms [70], serotonin has also been shown to play a role in the response to novelty-seeking [71], [72].

Although we have previously reported that intermittent EtOH exposure during adolescence does not modify spontaneous behavior (anxiety and social behavior) in adult mice [12], in the present study an effect of this pattern of EtOH administration was evident in HNS mice. The relationship between aggression and EtOH consumption has been demonstrated by numerous studies. In rats and mice, acute high doses of EtOH reduce aggressive behaviors [73], [74], whereas low doses can induce a slight increase in aggression [75], [76]. On the other hand, subordinate males consume much more EtOH than dominant ones [75]. Administration of EtOH during pregnancy or the postnatal period reduces aggression in the adult progeny [77]. Aggression frequently coincides with specific dimensions of emotionality, such as impulsivity, risk-taking and drug abuse. Although previous reports [78] have shown aggressive behaviors to be modulated by the level of novelty-seeking, significant differences were not found between HNS and LNS saline-exposed mice in the present study. In a previous report we have shown that a similar pattern of EtOH exposure during adolescence produces a slight decrease in aggressive behaviors in adult mice [12], but in the present study this effect was significantly present only in HNS mice.

Human and animal studies have shown that acute exposure to low-to-moderate doses of ethanol induces anxiolytic-like behavior [40]. Thus, the anxiolytic effects of alcohol could be involved in the reinforcing properties of the drug: individuals with chronic stress or high anxiety levels may be more sensitive to the anxiolytic effects of ethanol and more predisposed to consume alcohol than unstressed subjects. High-anxious mice exhibit higher consumption and preference for ethanol than low-anxious counterparts, but not for saccharin and quinine, suggesting alterations in the rewarding effects of alcohol [79]. On the other hand, Conrad and Winder [80] found that stressed adolescent mice exposed to EtOH displayed lower levels of anxiety-like behavior than adult animals in the EPM. Considered together, these findings suggest a correlative link between trait anxiety and behavioral responses to ethanol. In line with this, we have observed that EtOH induced an intense anxiolytic effect in HNS animals only. However, it should be noted that our control group was composed of saline-treated mice that were also exposed to repeated injections, which are stressful in themselves. This stress could have affected the anxiety profile of the saline-treated mice, which only spent between 10 and 15 percent of their time in the open arms of the maze, a much lower percentage than that reported by previous studies performed under similar conditions [12].

For measuring the effects of EtOH administration on novelty seeking, the two novelty-seeking tests used in the present study provided the mice with free-choice novelty, but the novel object recognition task involves recognition memory, detection and processing of the novel object [81]. In the novel object recognition task, no significant differences were detected between HNS and LNS in the group exposed to saline. Animals catalogued as HNS depending on the number of DIPS they made in the first hole board test did not devote a higher percentage of time to exploring the novel object or perform more explorations. Similar results were observed in the second HB test, in which mice catalogued as HNS during adolescence performed the same number of DIPS as LNS in the second HB. This decrease in novelty seeking could have been due to the differing age of the mice, as the first HB test was performed on PND 31, while the second HB test and the novel object recognition task were performed on PND 69 and 70, respectively. Administration of EtOH significantly decreased exploration of the novel object, and the higher EtOH dose also reduced the number of explorations of the novel object in both types of animals. In contrast with these results, EtOH administration did not affect the number of Dips in the second HB test. Our results differ with those of Stansfield and Kirstein [41], who observed that rats chronically exposed to ethanol during adolescence and tested in adulthood made more approaches towards the novel object, suggesting that exposure to ethanol during development results in less-inhibited behavior during adulthood. These contradictory results may be the result of employing different species and methodologies: for instance, the study of Stansfield and Kirstein included more habituation trials and the test lasted only 5 minutes. In line with our results, a more recent study found that withdrawal from repeated treatment with ethanol induced a protracted decrease in novelty-seeking behavior, which the authors attributed to the development of anhedonia [82]. Although that study employed the novelty place preference test to evaluate novelty-seeking, it was similar to ours in that the same species was employed and the test sessions had the same as ours.

Novelty-seeking appears to be a risk factor for drug addiction in general, as it tends to increase the risk of early experimentation with drugs. Moreover, initial exposure and repetitive use of drugs may also be influenced by personality risk factors. In adolescents, traits related to positive emotionality and approach behavior, such as sensation-seeking, have been associated with alcohol use [83]. The present study demonstrates that the novelty-seeking phenotype also influences the long-lasting effects of chronic EtOH administration. In general, HNS adolescents have their first contact with drugs earlier than their LNS peers, and the results of our study suggest that such exposure to drugs induces profound changes in their future behavior and modifies their response to other drugs (e.g. cocaine). Further knowledge of how personality factors modify the effects of drugs of abuse is vital for drawing up effective guidelines for preventive strategies.
